# Malaria therapeutics: are we close enough?

**DOI:** 10.1186/s13071-023-05755-8

**Published:** 2023-04-14

**Authors:** Himani Tripathi, Preshita Bhalerao, Sujeet Singh, Hemant Arya, Bader Saud Alotaibi, Summya Rashid, Mohammad Raghibul Hasan, Tarun Kumar Bhatt

**Affiliations:** 1 Department of Biotechnology, Central University of Rajasthan, NH-8, Bandarsindri, 305817 Rajasthan India; 2 Department of Clinical Laboratory Science, College of Applied Medical Sciences, Alquwayiyah, Shaqra University, Riyadh, 11971 Saudi Arabia; 3 Department of Pharmacology and Toxicology, College of Pharmacy, Prince Sattam Bin Abdulaziz University, P.O. Box 173, Al-Kharj, 11942 Saudi Arabia

**Keywords:** Diagnostics, Malaria therapeutics, *Plasmodium species*, Rapid diagnostic test, RTS,S, Vaccine

## Abstract

Malaria is a vector-borne parasitic disease caused by the apicomplexan protozoan parasite *Plasmodium*. Malaria is a significant health problem and the leading cause of socioeconomic losses in developing countries. WHO approved several antimalarials in the last 2 decades, but the growing resistance against the available drugs has worsened the scenario. Drug resistance and diversity among *Plasmodium* strains hinder the path of eradicating malaria leading to the use of new technologies and strategies to develop effective vaccines and drugs. A timely and accurate diagnosis is crucial for any disease, including malaria. The available diagnostic methods for malaria include microscopy, RDT, PCR, and non-invasive diagnosis. Recently, there have been several developments in detecting malaria, with improvements leading to achieving an accurate, quick, cost-effective, and non-invasive diagnostic tool for malaria. Several vaccine candidates with new methods and antigens are under investigation and moving forward to be considered for clinical trials. This article concisely reviews basic malaria biology, the parasite's life cycle, approved drugs, vaccine candidates, and available diagnostic approaches. It emphasizes new avenues of therapeutics for malaria.

**Graphical Abstract:**

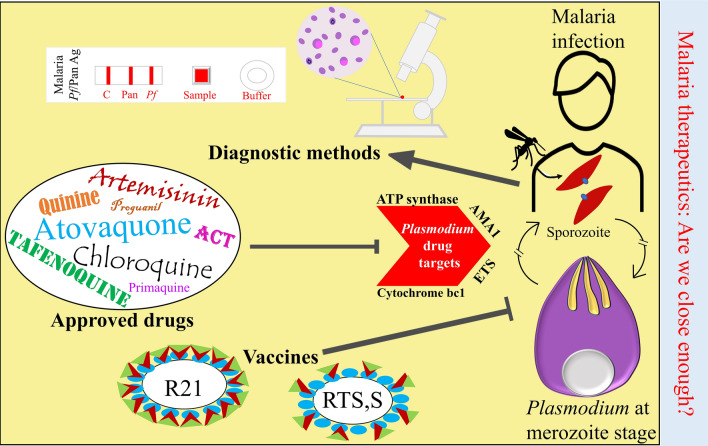

## Background

Malaria is one of the most severe and life-threatening diseases. It is a mosquito-transmitted infectious disease and a major global health issue in tropical and subtropical regions. The mortality rate of malaria is very high compared to other protozoan diseases. As per the World Health Organization (WHO) 2020 reports, 241 million malaria cases were recorded worldwide; 627,000 people died from malaria. WHO announced the Global technical strategy (GTS) 2016–2030 to eradicate malaria by reducing malaria case incidence and mortality rates by at least 90% [1]. Malaria elimination depends on (i) preventive measures (including vaccination) and vector control, (ii) a sensitive diagnostic technique, and (iii) proper treatment of malaria infection on time [2]. Malaria is categorized as (i) asymptomatic malaria (caused by most of the *Plasmodium* species; infected individuals exhibit no symptoms or clinical signs), (ii) uncomplicated malaria (caused by human infecting *Plasmodium* species; symptoms include fever, moderate to severe body shaking, chills, sweating, anemia, vomiting, and nausea), and (iii) severe malaria (mainly caused by *Plasmodium falciparum*; symptoms include severe anemia, multiple organ failure, coma in case of cerebral malaria, pulmonary complications, acute kidney-associated injury, blood coagulation problems, metabolic acidosis, high temperature of 39 to 41 ºC, polyuria, and myalgia). Malaria is often fatal when not diagnosed and treated in a timely manner [3].

**Fig. 1:**
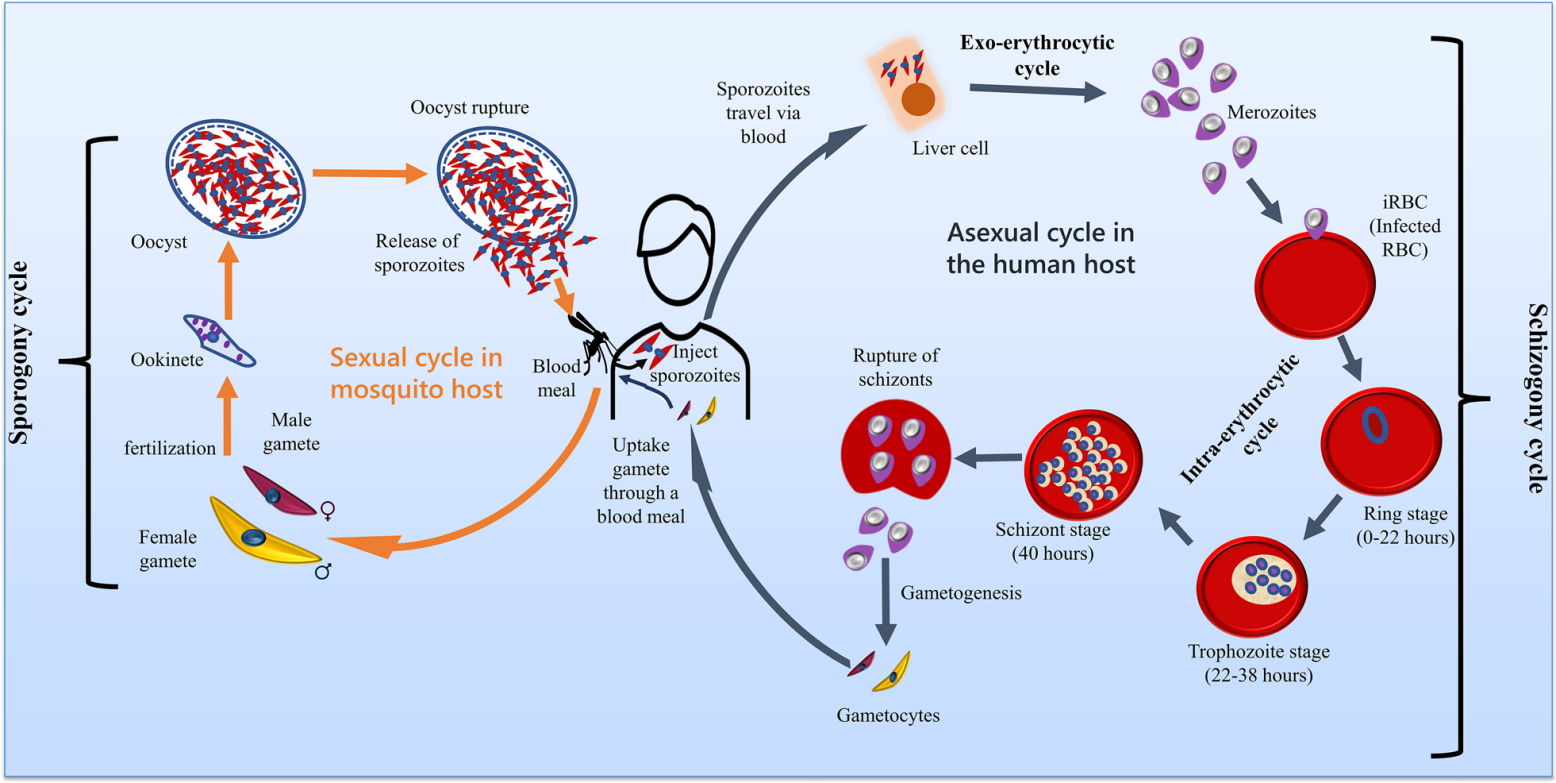
Life cycle of malaria parasite

Five different species of *Plasmodium* (*P. falciparum*, *P. vivax*, *P. ovale*, *P. malariae*, and* P*. *knowlesi*) cause malaria infection in humans. The parasite is infective and motile to the vertebrate host, and the malaria parasite life cycle involves two hosts (digenetic): human (intermediate host) and female *Anopheles* mosquito (definitive host) [3]. When an *Anopheles* mosquito bites a healthy human being, it injects sporozoites (infective stage for humans) while sucking the blood meal. These sporozoites are transferred to the hepatic cells (in the liver) through the blood circulatory system. These sporozoites mature into merozoites (exo-erythrocytic cycle) inside the hepatic cells. They are released into the blood vessel and invade the erythrocytes in which they grow and re-invade the fresh red blood cells (RBCs) for the completion of the erythrocytic cycle (asexual stage) (Fig. 1) [3–5].

In the erythrocytic cycle, merozoites convert into ring-stage trophozoites, further developing into mature trophozoites and then into schizonts, and this development cycle takes around 48 h for *P. falciparum*. Later, schizonts rupture and release 8 to 36 merozoites to invade the new RBCs and continue the cycle where some of the merozoites undergo sexual development and mature into the male and female gametocytes (infective stage for mosquito); this process is called gametocytogenesis [6]. These gametocytes are taken by mosquitos while sucking the blood meal. Inside the mosquito gut, the microgamete (male gamete) fuses with the macrogamete (female gamete) and produces a zygote (gametogenesis) [6]. The zygote matures into ookinetes, which takes approximately 24 h. The ookinetes then develop into oocysts; this maturation occurs between the epithelium and basal lamina of the mosquito gut. The oocysts grow and rupture, releasing sporozoites (formed by asexual replication). The sporozoites are released and migrate through the hemocoel; they invade and are stored in the salivary gland of mosquitoes (Fig. 1).

Malaria is one of the major global health problems and annually causes significant mortality, morbidity, and socioeconomic burden. In the last 20 years, the world has achieved enormous progress in eliminating malaria. Timely diagnosis and effective treatment can prevent human health from diseases, including malaria [7, 8]. Several methods, such as rapid diagnostic test (RDT), microscopy-based analysis, and thick and thin layer blood smear analysis, are available to diagnose malaria. RDT is an accurate, quick, and WHO-recommended approach for identifying persons with symptomatic malaria and high parasite counts, and it is also used outside health institutions (remote areas) where a sophisticated lab is not established for malaria testing [9]. The RDTs are very affordable and straightforward to use. There is no requirement for a trained person. The available malaria diagnostic methods are explained in this review article.

A therapeutic approach can be preventive or curing. A vaccine is the most trusted preventative measure to control many infectious diseases. A vaccine is vital in eliminating any disease because it provides sterile lifelong immunity [8]. WHO recently approved RTS,S vaccine to cure malaria. The RTS,S vaccine targets the *P. falciparum* circumsporozoite surface protein (PfCSP) and shows < 37% protection against the *P. falciparum* parasite in the third phase of clinical trials [6, 10]. Several malaria vaccines targeting various stages of the parasite’s life cycle have been tried, but vaccine development against the eukaryotic malarial parasite is still very challenging. This review also discusses the potential malarial vaccines [11]. In addition, the availability of effective drugs for any disease may lower the overall mortality rate. Commonly used antimalarials are chloroquine, amodiaquine, quinine, mefloquine, halofantrine, lumefantrine, primaquine, and atovaquone. These drugs provide first-line protection to malaria patients. Moreover, WHO recommended artemisinin-based combination therapies (ACTs) as the first- and second-line treatment for the uncomplicated *P. falciparum* and chloroquine-resistant *P. vivax* malaria [12, 13]. In the ACT approach, artemisinin is combined with its derivatives, such as artesunate, dihydro-artemisinin, and artemether, and given to malaria patients for their betterment. Unfortunately, the malaria parasites are becoming resistant to the existing anti-malarial drugs, so there is an urgent need to develop or identify new potential drug/s against the malaria parasite [9, 13, 14]. This review article touches upon all the aspects of malaria therapeutics, explaining available therapeutics (vaccine/s and drug/s), diagnostic methods, and possible advancement in the respective fields.

### Antimalarial drugs and their targets

**Fig. 2:**
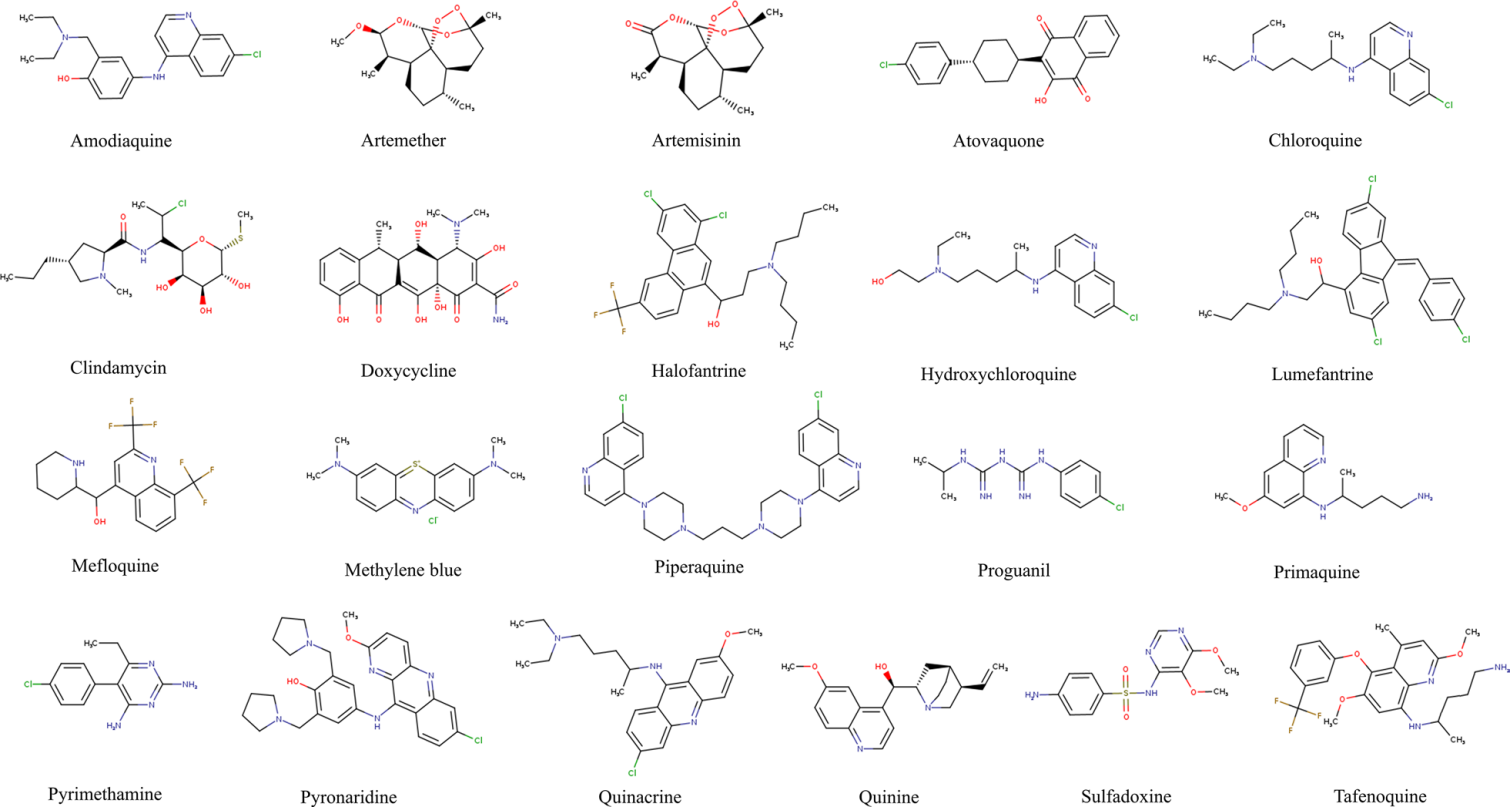
List of approved antimalarials

Malaria is a severe global health problem that can lead to death if not treated in a timely manner. A drug is a chemical substance (natural or synthetic) used to diagnose, prevent, treat, and cure a disease [13]. Several antimalarial drugs are used worldwide to treat and prevent malaria infection [15]. Antimalarials are divided into several classes based on quinoline (4 and 8 aminoquinolines), cinchona alkaloids, diaminopyrimidine, sulfonamides, tetracyclines, naphthoquinones, and sesquiterpenes [16, 17]. Antimalarials are used as prophylaxis for malaria, and most kill parasites in the infected RBCs. Unfortunately, mosquitoes are becoming resistant to most of the approved antimalarials. Many have severe side effects such as blurred vision, stomach upset, nausea, vomiting, insomnia, headache, loss of appetite, hair loss, and mood swings [18]. One of the focuses of this review paper is to list all the anti-malarials and their drug targets (Table 1 and Fig. 2).

**Table 1:** List of antimalarial drugs along with their drug targets and site of action

Sl. no	Drug	Drug target/s and mechanism	Site of action	Refs.
1	Amodiaquine	Not known, but it is assumed that it inhibits heme polymerase activity. The amodiaquine-heme complex is toxic and disrupts the membrane function of the parasite	Food vacuole	[19]
2	Artemether	Inhibits nucleic acid and protein synthesis at erythrocytic stages of *Plasmodium falciparum*	Food vacuole	[20]
3	Artemisinin	Sarco/endoplasmic reticulum Ca^2+^-ATPase (SERCA) of *P. falciparum* Alkylation of essential malarial proteins and lipids	ER, vesicular structures	[21]
4	Atovaquone	Cytochrome bc1 complex (Complex III). Inhibits the parasite mitochondrial ETC pathway, resulting in the loss of mitochondrial function	Mitochondria	[22]
5	Chloroquine	Chloroquine inhibits hemozoin formation, leading to the accumulation of heme in the food vacuole. The free heme then lyses the membrane and leads to parasite death Inhibits DNA and RNA synthesis	Food vacuole	[23]
6	Clindamycin	Clindamycin is a lincosamide antibiotic that exerts antimalarial activities (against multidrug-resistant *P. falciparum*) when given in combination with quinine. It inhibits protein synthesis	-	[24]
7	Doxycycline	Doxycycline (an antibiotic) inhibits 30S ribosomal translation inside the essential apicoplast organelle, leading to parasite death It is also given with quinine	Apicoplast	[25]
8	Halofantrine	Halofantrine appears to inhibit heme polymerization, resulting in the parasite being poisoned by its waste It also acts as a blood schizonticide	RBC	[26]
9	Hydroxychloroquine	The exact mechanism is unknown. It is assumed that hydroxychloroquine acts similarly to chloroquine. In addition, it accumulates in the parasite lysosomes. It raises the vacuole's pH, leading to essential protein degradation and affecting the post-translation modification of proteins in the Golgi bodies	Food vacuole	[27]
10	Lumefantrine	The exact mechanism is unknown. It is assumed that it inhibits β-hematin formation by forming a hemin complex and inhibits protein and nucleic acid synthesis It also acts as a blood schizonticide and exerts effects against erythrocytic stages of *Plasmodium spP*	RBC	[28]
11	Mefloquine	The action of the mechanism is not entirely understood. A few reports suggest that it inhibits parasite protein synthesis through direct binding to the cytoplasmic ribosome (80S-ribosome) of *P. falciparum*, which leads to cause schizonticidal effects It damages the parasite’s membrane	Ribosome	[29]
12	Methylene blue	It inhibits *P. falciparum* glutathione reductase, which hampers the polymerization of heme into hemozoin (essential for parasite survival)	RBC	[30]
13	Piperaquine	The mechanism is similar to chloroquine. It inhibits the heme detoxification pathway of *P. falciparum*	Food vacuole	[31]
14	Primaquine	The mechanism is unclear. It interferes with the mitochondrial ETS pathway and destroys mitochondria	Mitochondria	[32]
15	Proguanil	Proguanil inhibits dihydrofolate reductase of the *Plasmodium*, which blocks the purine and pyrimidine biosynthesis. This inhibition leads to nuclear division failure at schizont formation in the liver and erythrocytes Inhibition of DNA synthesis	RBC, liver malaria: the past and the present	[33]
16	Pyrimethamine	Pyrimethamine inhibits dihydro-folate reductase (DHFR). The mechanism is similar to proguanil	RBC, liver	[34]
17	Pyronaridine	Under investigation. It is an erythrocytic schizonticide It binds with DNA and disturbs nucleic acid metabolism	RBC	[35]
18	Quinacrine	Under investigation. It binds with DNA and disturbs nucleic acid metabolism	RBC	[36]
19	Quinine	Purine nucleoside phosphorylase enzyme (inhibits the spontaneous formation of hemozoin) It inhibits protein synthesis and glycolysis. It acts as a blood schizonticide and has gametocytocidal activity against *Plasmodium vivax* and *P. malariae*	Digestive vacuole	[37]
20	Sulfadoxine	Sulfadoxine targets *Plasmodium* dihydropteroate synthase (it converts para-aminobenzoic acid to folic acid, which helps in nucleic acid synthesis) and dihydrofolate reductase (DHFR) proteins	Schizonts	[38]
21	Tafenoquine	The mechanism is not well established. It is assumed that it also inhibits heme polymerase (resulting in the parasite being poisoned by its waste) in the blood stage of the parasites	RBC	[39]

**Fig. 3:**
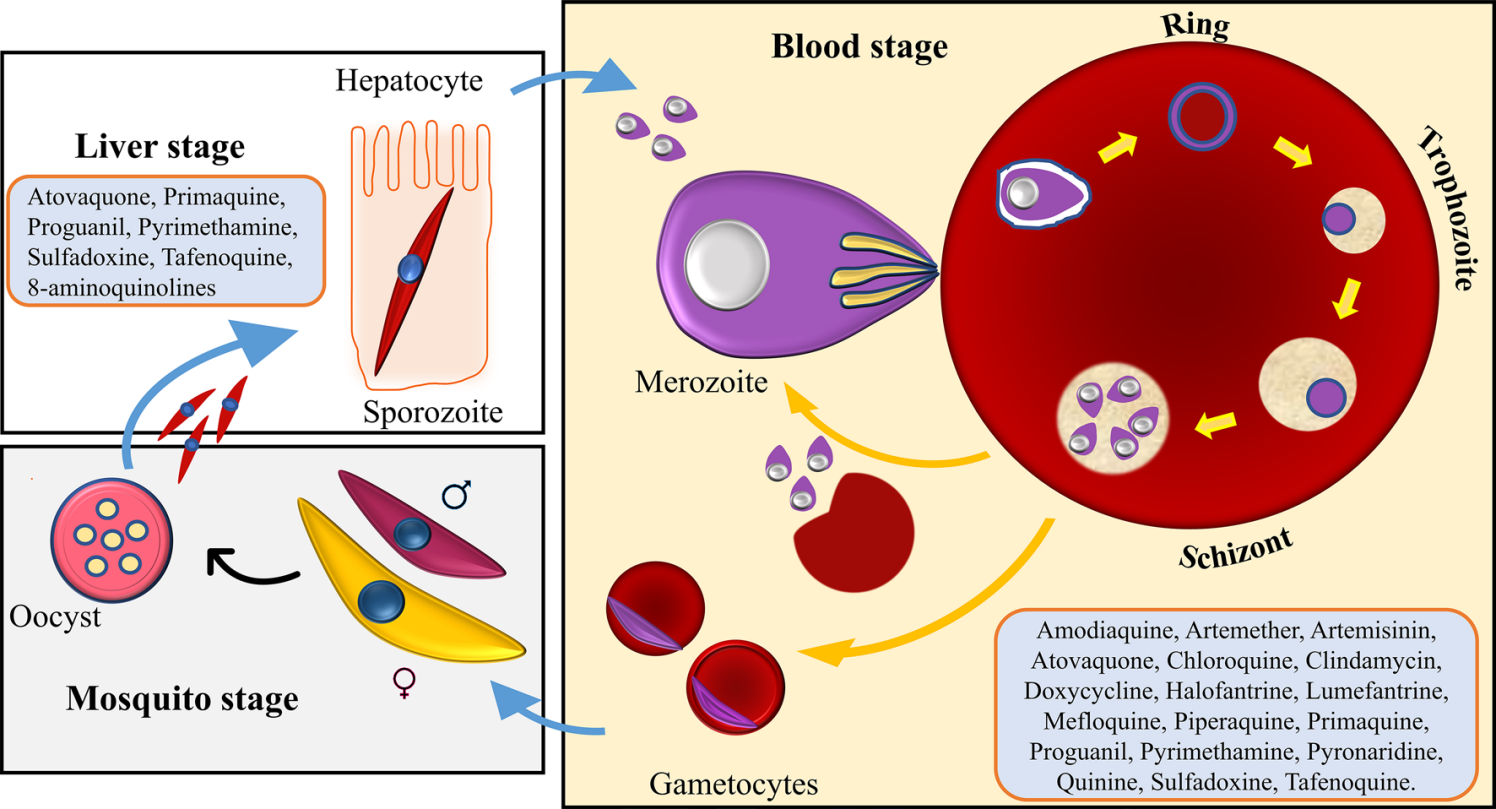
Antimalarials targeting the parasite’s life cycle

Primarily, drugs interact with biological macromolecules (protein/DNA/RNA) in the human body to alter the physiological function and produce the desired effect. Drugs perform specific acts by interfering with macromolecules and altering cellular or biochemical processes, often called ‘drug targets.’ The structure and biochemistry of the *Plasmodium* parasite were studied very well, leading to understanding and identifying potential drug targets to overcome malaria. Malarial drug targets are classified based on their location in the parasite (e.g. food vacuole, apicoplast, cytosol, membrane, and mitochondria) and their mechanism (e.g. heme polymerization, pyrimidines de novo synthesis, DNA/protein synthesis, TCA cycle, ETC pathway, membrane transport, and hemoglobin hydrolysis). The essentiality of any protein in parasite development makes them potential drug targets [40, 41]. Table 2 and Fig. 3 collect all the reported drug targets for malaria disease.

**Table 2:** List of potential antimalarial targets

Sl. no	Drug target	Functions in parasite	Refs.
1	Acetyl CoA synthetase	It provides acetyl moieties for several metabolic and regulatory cellular pathways, which help in parasite growth	[42]
2	Adenylyl cyclase-β	It helps in cyclic adenosine monophosphate (cAMP) synthesis, an essential element of the parasite life cycle	[43]
3	Aminoacyl-tRNA synthetase	It helps in the protein synthesis (translation) process by adding an aminoacyl group to the 3’ end of tRNA	[44]
4	Aminopeptidase	It is a protease enzyme essential in cell maintenance, cell growth, peptide catabolism (during the asexual erythrocytic replication cycle), protein maturation, antigen presentation on immune cells, and hormone regulation	[15]
5	Anion-exchange protein-1	Involved in erythrocyte invasion (adhesion of infected erythrocytes to endothelial cells) and acid-base homeostasis regulation	[15]
6	Apical membrane antigen-1	Transmembrane protein. It helps parasites invade host erythrocytes	[15]
7	Aspartate aminotransferase	It is one of the key enzymes in energy metabolism and de novo biosynthesis of pyrimidines	[45]
8	Aspartate transcarbamoylase	It is essential in catalyzing the second step in de novo pyrimidine biosynthesis	[46]
9	ATP synthase	Involved in the ATP generation during aerobic glycolysis at the blood stage of the parasite’s life cycle	[47]
10	Calcium-dependent protein kinase-I	Significant role in activating translation of repressed mRNA during the sexual stage. It regulates parasite mobility. It helps in microneme secretion during erythrocyte invasion	[48]
11	Carbamoyl phosphate synthetase-II	It helps in the de novo pyrimidine biosynthesis process by catalyzing the formation of carbamoyl phosphate, glutamine, and ATP	[49]
12	Carbonic anhydrasU + 0065	Metalloenzyme. An essential metabolic enzyme catalyzes carbon dioxide's reversible conversion to bicarbonate in pyrimidines de novo synthesis	[15, 50]
13	Casein kinase 2α	Involved in the critical cellular process; cell differentiation, proliferation, stress response, DNA damage, apoptosis, and circadian rhythm	[51]
14	cGMP-dependent protein kinase	Key regulator of cGMP signaling in the malaria parasite. It is required in the parasite life cycle's sexual and asexual stages	[52]
15	Choline transporter	The first enzyme of the Kennedy pathway. involved in the biosynthesis of phospholipid and phosphatidyl-choline	[53]
16	Choline phosphate cytidylyl transferase	It catalyzes the rate-limiting step of the Kennedy pathway and is crucial for the survival of the murine parasite	[54]
17	Cysteine protease	Key role in hemoglobin degradation and erythrocyte cytoskeletal proteins hydrolysis	[15, 55]
18	Cytochrome bc1	Essential for pyrimidine biosynthesis	[47]
19	Cytochrome c oxidase (Complex IV)	Involved in *Plasmodium* mitochondrial electron transport chain	[56]
20	Dihydrofolate reductase	Involved in the pyrimidine synthesis in *Plasmodium* by de novo pathway	[57]
21	Dihydroorotate dehydrogenase	Involved in the de novo pyrimidine synthesis, the primary energy source and essential for parasite survival	[47]
22	Dihydropteroate synthase	Significant role in folate metabolism	[58]
23	Dipeptidyl aminopeptidase	Present in food vacuole and cleaves dipeptides from amino termini of proteins/oligopeptides	[59]
24	DNA methyltransferases	Involved in the epigenetic mechanism	[60]
25	DXP reductoisomerase	An essential enzyme of the DXP/MEP pathway that triggers isoprenoid formation (required for the production of cholesterol, dolichols, and ubiquinones)	[61]
26	Falcipain	Endopeptidase. It involves host hemoglobin hydrolysis, erythrocyte invasion, and erythrocyte rupture	[62]
27	Farnesyltransferase	Involved in the blood stage of the parasite life cycle	[63]
28	Fumarate hydratase	A vital component of the tricarboxylic acid cycle of the *Plasmodium* parasite (helps in the interconversion of fumarate to malate)	[47]
29	Gamma-glutamylcysteine synthetase	An essential enzyme of glutathione biosynthesis	[15]
30	Geranylgeranyl pyrophosphate synthase	Key branchpoint enzyme in isoprenoid biosynthesis	[43]
31	Glutathione reductase	A flavoenzyme regenerates glutathione (an essential enzyme antioxidant defense against cell damage)	[64]
32	Glutathione S-transferase	Involved in cellular detoxification	[65]
33	Glycerol 3-phosphate dehydrogenase	Key glycolytic homotetrameric enzyme. Involved in vesicular transport and apical organelle biogenesis	[66, 67]
34	Heat shock protein 90	The most abundant chaperone in cells is responsible for cell cycle regulation and signal transduction	[68]
35	Hemozoin	A crystallized heme dimer. Sequestration of heme into hemozoin formation is an essential process for parasite development	[69]
36	Hexose transporter	Glucose uptake is mediated by the hexose transporter enzyme	[70]
37	Histone acetyltransferase	Involved in the acetylation of histone tails that causes localized chromatin relaxation and transcriptional activation of nearby genes	[71]
38	Histone acetyltransferase GCN5	Key role in the epigenetic mechanism. It controls erythrocyte invasion and virulence in the *Plasmodium* parasite	[72]
39	Histone deacetylase	It catalyzes the deacetylation of acetylated histones leading to transcriptional repression	[71]
40	Hypoxanthine guanine phosphoribosyl transferase	Helps in DNA/RNA synthesis via de novo synthesis	[73]
41	Lactate dehydrogenase	The critical enzyme of energy production. It catalyzes the interconversion of pyruvate to lactate in the glycolysis process	[74]
42	Malate dehydrogenase	It involves NADH and citrate production to support the TCA cycle, ETC	[75]
43	Malatequinone oxidoreductase	Membrane protein. Involved in three essential pathways (ETC, TCA, and fumarate cycle)	[76]
44	Merozoite surface protein-1	Essential for the attachment of merozoites to host receptor. Role in RBC invasion	[15]
45	Methionine aminopeptidase 1b	Metalloproteases. Involved in protein maturation and activation by catalyzing the removal of the N-terminal initiator methionine during protein synthesis	[77]
46	Mitogen-activated protein kinase 2	It plays a vital role in critical cellular processes and signal transduction	[78]
47	N-myristoyl transferase	The key enzyme of post-translational modifications	[79]
48	NADH dehydrogenase type II	It is an essential enzyme of the *Plasmodium* mitochondrial electron transport chain system	[80]
49	Niemann-Pick Type C1	Present on the parasite’s plasma membrane—essential protein for the intraerythrocytic growth of *Plasmodium falciparum*	[81]
50	Ornithine decarboxylase	Involvement in polyamines biosynthesis (key component of transcription, translation, and several cellular processes)	[82]
51	Orotatephospho-ribosyl transferase	Crucial enzyme for the de novo pyrimidine synthesis pathway	[83]
52	Orotidine 5′-monophosphate decarboxylase	The key enzyme for the de novo pyrimidine synthesis pathway	[84]
53	Orphan protein kinase PfPK7	An essential enzyme in the melatonin transduction pathway	[85]
54	Pantothenic acid	Essential vitamin and precursor of coenzyme A	[86]
55	Phosphocholine cytidylyltransferase	Essential enzyme for the biosynthesis of phosphatidylcholine	[54]
56	Phosphodiesterase β	The key enzyme for developing the asexual blood stage of the malaria parasite	[87]
57	Phosphoinositide lipid kinases	Lipid phosphoinositides are signaling molecules involved in cellular functions (e.g. cell growth, cell division, and membrane trafficking)	[88]
58	Phosphatidylinositol 3-phosphate	An essential enzyme in vesicular trafficking processes and intraerythrocytic development	[89]
59	Phosphoribosyl transferase	Key purine salvage enzyme	[90]
60	Plasmepsin	Plasmepsin is responsible for hemoglobin digestion, cytoskeleton protein processing, oocyst development, parasite virulence modulation, and host-targeted protein export	[91]
61	Reactive oxygen species	It plays a significant role in the regulatory mediators in signaling processes (e.g. lipid peroxidation, cell signaling, ETC, and hemoglobin digestion)	[92]
62	Rhoptry-associated protein	Involve in the invasion of RBC by merozoites	[93]
63	S-adenosylhomo-cysteine hydrolase	It is required for the metabolic pathway (sulfur-containing amino acids) and biological methylation process	[94]
64	Serine repeat antigen-5	The key enzyme for parasite development at the blood stage	[95]
65	Serine/threonine-protein kinase	Involved in cellular processes such as differentiation, proliferation, cell cycle progression, apoptosis, and DNA damage	[96]
66	Signal peptide peptidase	Membrane-bound endopeptidases. Help in parasite protein maturation and transport	[97]
67	Subtilisin-like protease-1	Role in merozoite invasion and mediates the proteolytic maturation	[98]
68	Succinate dehydrogenase Complex II	Essential enzyme for the TCA, ETC pathway	[99]
69	SUMOylation	A post-translational modification enzyme in the parasite life cycle	[100]
70	Surface anion channel	Parasite-induced ion channel on host erythrocyte membrane mediates nutrients update, various bulky organic solutes, and supports intracellular parasite growth	[101]
71	Thioredoxin reductase	Homo dimeric protein. It maintains redox equilibrium in the glutathione system	[102]
72	Thymidylate synthase	Involved in folate de novo synthesis	[103]
73	Topoisomerase	Role in DNA transcription, replication, repair, and cell division	[104]
74	V-Type H + ATPase	Regulate intracellular pH and plasma membrane potential. It is associated with chloride channels	[105]

### Malaria vaccine

The vaccine as a preventive measure or to generate herd immunity in the community is essential in eradicating any disease. Some WHO-approved or under clinical trial vaccines for protozoan diseases are Leish-F1 and ChAd63-KH vaccine (human leishmaniasis), Leishmune and CaniLeish (canine leishmaniasis), s48 vaccine (toxoplasmosis in sheep), Mosquirix or RTS,S (malaria) [106, 107]. RTS,S is the only malaria vaccine approved by WHO for its pilot implementation in the malaria-endemic regions. Despite several efforts, RTS,S could generate only 50% protection and a subsequent decline in efficacy after a four-dose regimen. Therefore, there is an urgent need to develop an improved, efficient, and potential vaccine against malaria. The increase in cases or deaths and drug-resistant strains is dismaying and demands a safe and immunogenic vaccine against malaria.

**Fig. 4:**
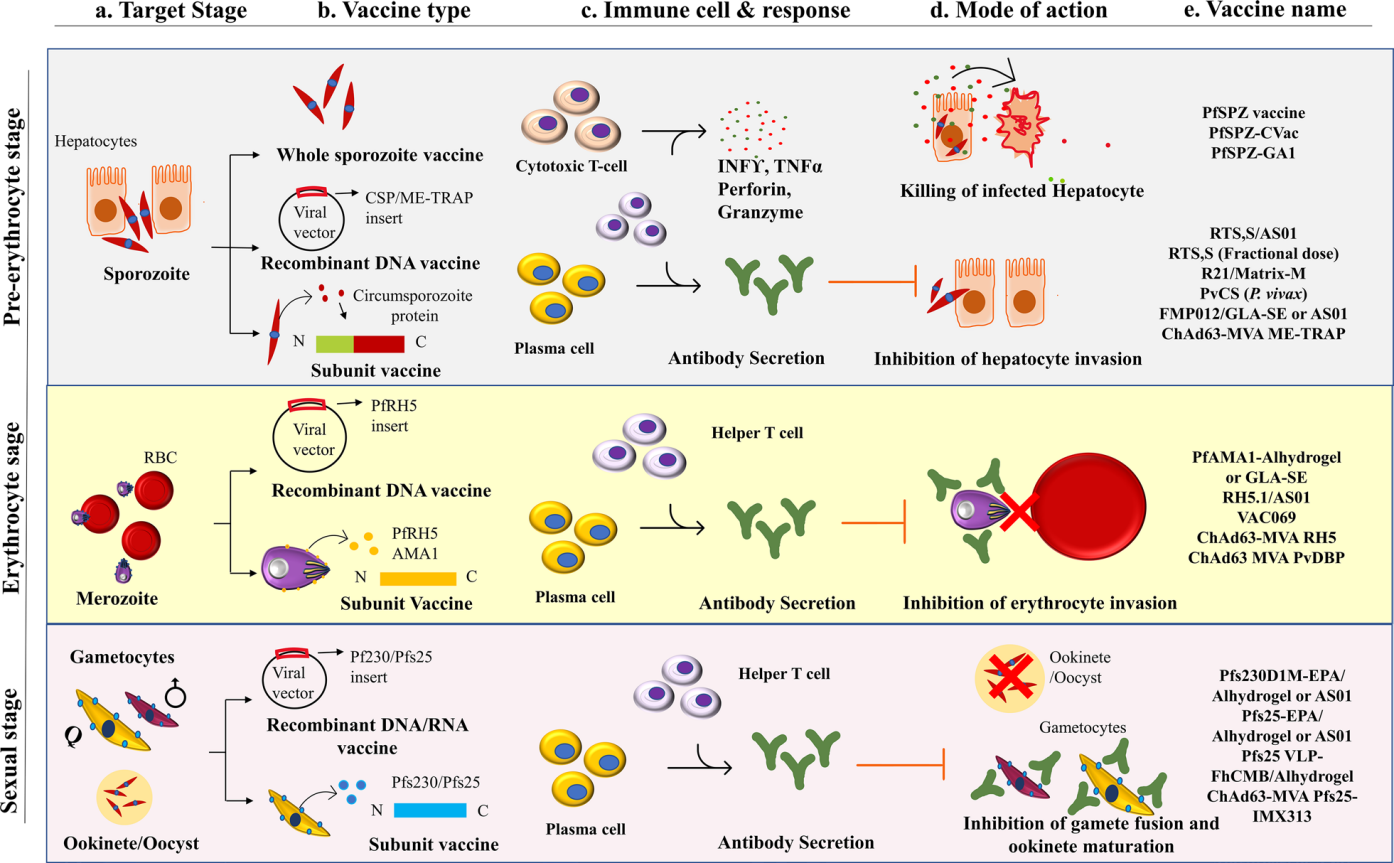
An overview of malaria vaccines and their mechanism. **a** Target stage (sporozoite, merozoites or gametocytes and ookinete). **b** Vaccine type (whole parasite vaccines, subunit vaccines, recombinant DNA/RNA vaccines) and target antigens. **c** Immune cell and response (cell-mediated or humoral antibody response). **d** Mode of action (killing of infected cell or cell invasion inhibition) of vaccine. (**e**) Vaccine name/s. DNA, deoxyribonucleic acid; RNA, ribonucleic acid

**Fig. 5:**
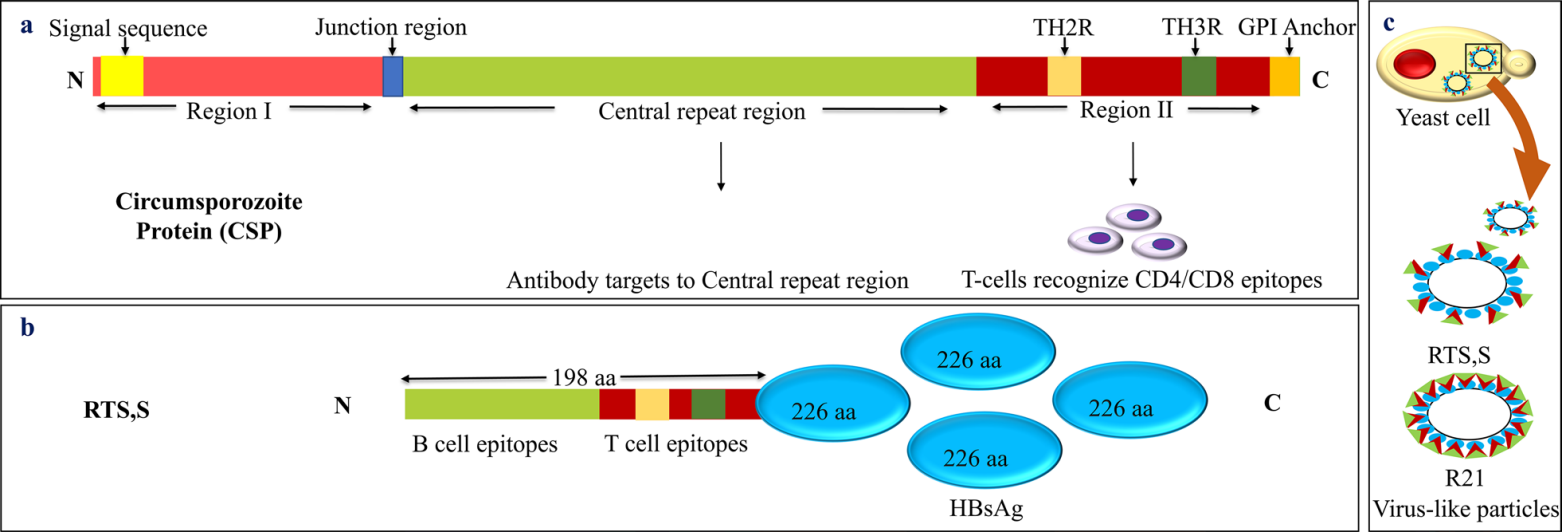
Schematic representation of (**a**) CSP region I and region II at N-terminal and C-terminal, respectively, and a central repeat region consists of NANP amino acid repeats. The junction region joins central repeat region to N-terminal and a signal sequence and GPI anchor sequence at N-terminal and C-terminal end, respectively. Central repeats (NANP) and junction region induce antibody generation while CD4 and CD8 T-cell epitopes at C-terminal trigger cell-mediated immune response. **b** RTS,S vaccine consists of B-cell epitopes from central repeat region at N-terminal and T-cell epitopes from region II of CS protein fused with hepatitis surface antigen (HBsAg) at C-terminal along with three copies of HBsAg (not in fusion with CS protein). **c** Yeast cell producing VLP (RTS,S and R21 vaccines) expressing CSP antigen on surface fused with hepatitis B surface antigen (HBsAg). GPI, glycosylphosphatidylinositol; CD, clusters of differentiation; CSP, circumsporozoite protein; VLP, virus-like particle

It has been more than 5 decades since Nussenzweig et al. used irradiated *Plasmodium berghei* sporozoite to immunize mice in their study and found that mice were protected when challenged with infectious sporozoite [108, 109]. However, mice were infected when challenged with blood-stage parasites, meaning only stage-specific protection was generated. An important lesson concluded from this study was to explore the parasite’s proteome, select essential protein/s from different stages of the *Plasmodium*’s life cycle, and develop stage-specific vaccine candidates to eradicate malaria worldwide. Stage-specific malaria vaccine could be produced by targeting the life-cycle stages such as (i) pre-erythrocyte stage, (ii) erythrocyte or blood stage, and (iii) sexual or transmission-blocking vaccine (TBV) (Fig. 4) [110]. Keeping this in mind, several proteins or biomolecules present on the surface of sporozoite, [circumsporozoite protein (CSP), cell-traversal protein for ookinetes and sporozoite (CELTOS)], blood-stage merozoite [merozoite surface protein (MSP), apical membrane antigen 1 (AMA1)], gametocyte (Pfs230), and ookinete (Pfs25 or Pvs25) have been characterized [111]. For instance, the identification of the CSP and its characterization has created a path ahead for malaria vaccine development [112]. CSP found abundantly on the sporozoite surface is the most potent candidate due to its high immunogenicity. It has a central repeat sequence [Asn-Ala-Asn-Pro (NANP) in *P*. *falciparum*], N-terminal region I, and a C-terminal region II which show similarity to thrombospondin and other adhesive proteins (Fig. 5) [113]. However, there is a distinct difference (sequence heterogenicity) between the repeat region of *P. vivax* VK210 (Type 1) and VK247 (Type 2) strains [114]. A chimeric construct targeting conserved C-terminal and repeat regions from these diverge strains would be suitable against pre-erythrocyte stage infection and prevent the hypnozoite dormant stage of *P. vivax* [115]. A junction between N-terminal and central repeats was recognized by antibodies specific to NANP repeats. The dual-capacity antibody can bind to the junction region and the NANP region and is characterized as the most potent among other antibodies against PfCSP [116]. Several vaccines have been designed and developed taking CSP as an antigen and tested in mice models [117]. A trend of subunit vaccines has been set employing NANP repeats and the C terminal region of CSP antigen for a malaria vaccine [118]. Further in this review, we will discuss the emerging candidates for the malaria vaccine (Table 3 and Fig. 4).

**Table 3:** List of malarial vaccines (approved or under clinical trial)

Candidate	Target stage	Vaccine type	Target antigen	Mode of action	Adjuvant	Phase/status	Clinical trial.gov identifier Home—ClinicalTrials.gov
PfSPZ	Pre-erythrocyte/liver-stage	Whole parasite	Sporozoite surface proteins	Antibodies block hepatocyte invasion or may kill infected hepatocytes	Not present	II Completed	NCT03952650
PfSPZ-GA1	Pre-erythrocyte/liver-stage	Whole parasite	Sporozoite surface proteins	Antibodies block hepatocyte invasion	Not present	I/IIa completed	NCT03163121
ChAd63-MVA ME-TRAP	Pre-erythrocyte/liver-stage	Recombinant DNA vaccine	Sporozoite surface protein 2 (TRAP)	Antibodies block hepatocyte invasion	MVA (Booster)	IIb Ia (intravenous) completed	NCT03707353
RTS,S	Pre-erythrocyte/liver-stage	Subunit	CSP	Antibodies block hepatocyte invasion	AS01	III/IV completed	NCT03143218
RTS,S (Fractional dose)	Pre-erythrocyte/liver-stage	Subunit	CSP	Antibodies block hepatocyte invasion	AS01	II active	NCT03276962
R21/Matrix-M	Pre-erythrocyte/liver-stage	Subunit	CSP	Antibodies block hepatocyte invasion	Matrix-M	IIb/III active	NCT04704830
R21/AS01B	Pre-erythrocyte/liver-stage	Subunit	CSP	Antibodies block hepatocyte invasion	AS01B	I/IIa	NCT02600975
FMP012 (PfCELTOS)	Pre-erythrocyte/liver-stage	Subunit	Cell-traversal ookinete and sporozoite protein	Inhibits sporozoite traversal through mosquito midgut and salivary gland	GLA-SE/ AS01	I/IIa completed	NCT01540474
PvCS	Pre-erythrocyte/liver-stage	Subunit	*Plasmodium vivax* CSP	Blocks hepatocyte infection	Montanide ISA	II active	NCT04739917
EP1300 polyepitope	Pre-erythrocyte/liver-stage	Vectored	CSP, TRAP, LSA-1, Exported protein 1 (EXP-1)	Antibodies block hepatocyte invasion	pMB75.6	I completed	NCT01169077
ICC-1132	Pre-erythrocyte/liver-stage	Subunit	CSP	Antibodies block hepatocyte invasion	Alhydrogel	I completed	NCT00587249
PEV3A virosome FP9-MVA ME-TRAP	Pre-erythrocyte/liver-stage	Vectored	TRAP	Antibodies block hepatocyte invasion	FP9-MVA	I/IIa completed	NCT00408668
PfCS 102	Pre-erythrocyte/liver-stage	Subunit	Sporozoite protein	Antibodies block hepatocyte invasion	Montanide ISA 720	I completed	NCT01031524
FP9 CS and MVA CS	Pre-erythrocyte/liver-stage	Vectored	CSP	Antibodies block hepatocyte invasion	FP9-MVA	I completed	NCT00121771
PEV301 and PEV302	Pre-erythrocyte/liver-stage	Subunit	CSP and AMA1	Antibodies block hepatocyte or merozoite invasion	Influenza virosome IRIV	Ib completed	NCT00513669
PEBS-POC1	Pre-erythrocyte/liver-stage	Subunit	Synthetic protein containing 131 amino acid	Antibodies block hepatocyte invasion	Aluminium Hydroxide	I/IIa	NCT01605786
FMP011	Pre-erythrocyte/liver-stage	Subunit	LSA1	Antibodies block hepatocyte invasion	AS01B	I/IIa completed	NCT00312663
ChAdOx1 LS2 and MVA LS2	Pre-erythrocyte/liver-stage	Vectored	LSA1 and LSAP2	Antibodies block hepatocyte invasion	Viral vector	I/II completed	NCT03203421
DNA Ad	Liver and bloodstage	Vectored	CSP and AMA1	Antibodies block hepatocyte and merozoite invasion	Adenovirus type 5 (Booster)	I/IIa completed	NCT00870987
ChAd63-MVA RH5	Blood stage	Vectored	Reticulocyte-binding protein homolog 5	Inhibits merozoite invasion	MVA (Booster)	Ia/Ib completed	NCT03435874
RH5.1	Blood stage	Subunit	Reticulocyte-binding protein homolog 5	Inhibits merozoite invasion	AS01	I/IIa Completed	NCT02927145
P27A	Blood stage	Subunit	Pf27	Inhibits merozoite invasion	Alhydrogel / GLA-SE	Ia/Ib completed	NCT01949909
ChAd63 MVA PvDBP	Blood stage	Vectored	Duffy binding protein	Inhibits merozoite invasion	MVA (Booster)	II active	NCT04009096
PvDBPII	Blood stage	Subunit	Duffy binding protein	Inhibits merozoite invasion	Matrix M1	I/IIa active	NCT04201431
VAC069	Blood stage	Whole parasitized/iRBC	Blood stage antigens	It inhibits merozoite invasion and may kill infected erythrocytes	Not present	I active	NCT03797989
RH5.1	Blood stage	Subunit	Reticulocyte binding protein homolog 5	Inhibits merozoite invasion	Matrix-M	Ib	NCT04318002
MSP3-CRM-Vac4All	Blood stage	Subunit (conjugate protein)	MSP 3	Inhibits merozoite invasion	Alhydrogel	I active	NCT05197751
AdCh63 MSP1 and MVA-MSP1	Blood stage	Vectored	MSP1	Inhibits merozoite invasion	Viral vector	I/IIa completed	NCT01003314
GMZ2	Blood stage	Vectored	Glutamate-rich protein (GLURP) and MSP3	Inhibits merozoite invasion	Aluminum hydroxide	I	NCT00424944
MSP3-LSP	Blood stage	Subunit	MSP3	Inhibits merozoite invasion	Aluminium hydroxide	IIb completed	NCT01341704
PAMVAC Placental malaria vaccine	Blood stage	Subunit	VAR2CSA	Inhibits merozoite invasion	Alhydrogel or GLA-LSQ or GLA-SE	I completed	NCT02647489
FMP2.1	Blood stage	Subunit	AMA1	Inhibits merozoite invasion	AS02A	II completed	NCT00460525
EBA-175 RII-NG	Blood stage	Subunit	EBA-175 region II	Inhibits merozoite invasion	Aluminium Phosphate	I completed	NCT00347555
AMA1-C1	Blood stage	Subunit	AMA1	Inhibits merozoite invasion	Alhydrogel + CpG 7909	I completed	NCT00344539
ChAd63 RH5	Blood stage	Vectored	RH5	Inhibits merozoite invasion	MVA RH5 (Booster)	Ia	NCT02181088
FMP2.1	Blood stage	Subunit	Merozoite protein AMA1	Inhibits merozoite invasion	AS01B	I/IIa completed	NCT02044198
FMP1	Blood stage	Subunit	MSP1	Inhibits merozoite invasion	AS02A	II completed	NCT00223990
AMA1-DiCo	Blood stage	Subunit	AMA	Inhibits merozoite invasion	GLA-SE/Alhydrogel	I completed	NCT02014727
MSP1(42)FVO and MSP1(42)3D7	Blood stage	Subunit	MSP1_42_	Inhibits merozoite invasion	Alhydrogel	I completed	NCT00340431
MSP1	Blood stage	Subunit	MSP1	Inhibits merozoite invasion	AS02A	I completed	NCT00317473
R0.6C	Sexual stage	Subunit	Pfs48/45	Generated antibodies block transmission activity	Matrix M/ Alhydrogel	I active	NCT04862416
Pfs25-IMX313	Sexual stage	Subunit	Pfs25	Generated antibodies block transmission activity	Matrix M	I active	NCT04271306
Pfs25 VLP-FhCMB	Sexual stage	Subunit	Pfs25	Generated antibodies block transmission activity	Alhydrogel	Ia completed	NCT02013687
ChAd63-MVA Pfs25-IMX313	Sexual stage	Vectored	Pfs25	Generated antibodies block transmission activity	MVA (booster)	Ia completed	NCT02532049
Pfs25-EPA	Sexual stage	Subunit	Pfs25 kDa ookinete protein	Generated antibodies block transmission to the mosquito	Alhydrogel/AS01	I completed	NCT01867463
Pfs230D1M-EPA	Sexual stage	Subunit	Pfs230 gametocyte protein	Transmission blocking activity. It may inhibit gametes fusion	Alhydrogel/AS01	II active	NCT03917654
Pfs25M-EPA + Pfs230D1M-EPA	Sexual stage	Subunit	Pfs25 and Pfs230	Transmission-blocking activity may inhibit gametes fusion	AS01	I completed	NCT02942277
PpPfs25 (*P. falciparum*) and ScPvs25 (*P. vivax*)	Sexual	Subunit	Pfs25	Generated antibodies block transmission activity	Montanide ISA51	I completed	NCT00295581
FP9 PP and MVA PP	Pre-erythrocyte	Vectored	Polyprotein		Viral vectors	I completed	NCT00374998
CS DNA MVA	Pre-erythrocyte/liver-stage	Vectored	CSP	Antibodies block hepatocyte invasion	Viral vector	Withdrawn	NCT00377494
p52-p36-GAP	Pre-erythrocyte/liver-stage	Whole parasite	Sporozoite proteins	Abs block hepatocyte invasion or may kill infected hepatocytes	Viral vector	I/IIa terminated	NCT01024686

### Whole parasite vaccine

After Nussenwieig’s research, several studies were performed by taking the whole parasite to develop a malaria vaccine using malaria-infected mosquitoes, irradiated by gamma radiation and allowed to feed on non-human primates and human subjects [108]. The idea was to mimic the natural infection. Complete protection was observed for weeks and months when challenged with infectious sporozoite or controlled human malaria infection (CHMI). Sanaria *P*. *falciparum* sporozoite (PfSPZ), a fast-track designated vaccine, has shown promising efficacy during its first and second clinical phase trial and is likely to move into the phase IIb/III trial. This non-replicating whole parasite was purified from irradiated mosquito's salivary glands and injected intravenously [119]. High-level protection was obtained against malaria up to 14 months after final immunization at a 9.0 × 10^5^ sporozoites dose when administered intravenously [120]. PfSPZ or whole parasite vaccine efficacy depends upon the number of sporozoites and dose regimen and the route of vaccine administration. Another method employed to use the live parasite is to inject PfSPZ along with anti-malarial drugs, e.g. chloroquine or pyrimethamine (termed as chemoprophylaxis vaccine PfSPZ-CVac), which kills the parasite and checks the intra-erythrocyte development cycle [121]. However, it does not affect the parasite’s liver stage development and continues to produce sporozoite, which triggers the immune response without causing the disease.

Since the PfSPZ-CVac has live sporozoite to induce an immune response, an increased fold of protection can be generated through this method compared with radiation attenuated sporozoite [122]. In parallel, PfSPZ-GA1, a genetically attenuated parasite (GAP), is a candidate vaccine (phase I/IIa) in which attenuation was obtained through genetic modification in the parasite genome [123]. GAP showcases all the sporozoite surface protein but fails to produce merozoites and prevent progression to the blood stage. In genetically attenuated vaccines, the modifications are being done by deletion of essential genes(s) (single, double, or triple gene knockout), resulting in loss of function or gain of function, or by overexpression of immunogenic protein or toxins (referred to as a suicidal parasite) [123–125]. Several researchers are trying to produce chimeric parasites that express the surface immunogen but cannot perform invasion activity. For example, the *P. falciparum* parasite expressing *P. vivax* CSP is a partial functional replacement of CSP and could be an excellent choice for whole parasite vaccine candidate as this chimeric parasite fails to generate infection [126]. The whole parasite or attenuated vaccine is always questioned for safety concerns due to incomplete attenuation and chances of revival of the parasite. Another major problem associated with the whole-parasite vaccine is the production of attenuated sporozoites (irradiated or genetically modified) and their storage and transportation, especially to the remote area of Saharan countries.

### Subunit vaccine

Subunit vaccine is the safest method of immunization with the most negligible probability of toxicity and reactogenicity. Also, the scale-up process of recombinant antigens is much easier than large-scale production and maintenance of attenuated parasites, making subunit vaccines a better choice for community immunization. Here are some examples of subunit vaccines for malaria, which are broadly categorized based on their target at various developmental stages of the parasite.

#### Pre-erythrocyte stage vaccine

RTS,S (Mosquirix), the first WHO-approved malaria vaccine, has set a milestone in WHO’s malaria eradication roadmap effort [127]. Based on virus-like particle (VLP) technology, RTS,S is a subunit vaccine having CSP antigen (NANP repeats and C-term region for *P. falciparum* vaccine) fused with hepatitis B surface antigen (HBsAg) on VLPs (Fig. 5) [128–130]. Four doses of RTS,S elicited short-lived protection that varied with different age groups and parasite strains [110]. R21/Matrix M, a subunit vaccine, has shown an increased efficacy compared with RTS,S in its second phase of clinical trials, employing the VLP technique and CSP antigen similar to RTS,S [131]. Unlike RTS,S, R21 does not contain hepatitis B surface antigen (HBsAg) in a separate form. HBsAg is expressed as a fusion protein with CSP antigen. In response to this fusion protein present on VLPs, most antibodies will be produced against the CSP and not against the HBsAg. This makes R21 (Rv21, *P. vivax* vaccine candidate) a promising vaccine candidate for malaria in the coming year [131]. However, high efficacy vaccine against malaria remains a challenge.

Various adjuvants have been tested with the CSP-based malaria vaccine for their efficacy, including AS02A, an oil-in-water-based adjuvant, and a polymeric glyco adjuvant p (Man-TLR7) conjugate with CSP, adenovirus 35/26 expressing CSP. The approved version of RTS,S vaccine for malaria, employed adjuvant system 01 (AS01) consisting of MPL A, a TLR4 agonist that induces biased Th1 response, and QS21, a highly purified saponin that does not work through only one such PRR or signaling cascade; instead, they enhanced antigen uptake and induced a strong Th1 and Th2 response [117, 132–135]. R21 formulated with different adjuvant Matrix M, a saponin-based adjuvant, has achieved 77% efficacy in clinical trials.

#### Erythrocyte or blood stage vaccine

MSPs present on the surface of erythrocytes infecting merozoites are the most studied blood-stage antigen(s) for drug and vaccine development among all *Plasmodium* species. This includes MSP1, MSP2, MSP3, MSP4, MSP8, and MSP10, characterized in parallel for vaccine development [136, 137]. MSP1, the prime target of antibody response of naturally acquired immunity during the parasite's blood stage, is often considered for the blood-stage vaccine. MSP1 is a high-molecular-weight protein (185 kDa) with multiple proteolytic cleavage sites. A C-terminal fragment of 42 kDa is found to be immunogenic and cleaves again into 33 kDa and 19 kDa fragments during invasion [138]. This 42 kDa (19 kDa fragment) fragment of MSP1 alone or in combination with other merozoite antigens (AMA1) was tested in clinical trials [136, 139]. Likewise, PvMSP1 (42 kDa) fused with PvMSP8 has been recently tested as a vaccine candidate against *P. vivax* infection [138]. Controlled blood-stage human malaria infection using inoculum of parasitized blood is a new approach to developing *P. vivax* vaccine. Currently, vaccine candidates targeting blood-stage which are under trials include ChAd63-MVA RH5 and MSP3-CRM-Vac4All.

Other vaccine candidates based on merozoite antigens for inhibition of erythrocyte invasion or clinical symptom progression are AMA1, RH5, SERA5, and PvDBP (*P. vivax* duffy binding protein), which measurably fail to generate a protective immune response [140]. Reticulocyte binding protein homolog 5 (PfRH5) can induce antibody response, which can inhibit parasite growth more efficiently than antibody response by PfMSP1 and PfAMA1, suggesting the critical role of RH5 in parasite growth and survival [11]. An effective delivery system and TLR-based adjuvants are required to enhance the immunogenicity of these polymorphic antigens. P27A is one such vaccine candidate that showed good immunogenicity in its first clinical trial and needed to be improved by considering an immunogenic delivery system [141].

#### Transmission blocking vaccine (TBV)

Candidates for TBV or sexual stage vaccine are Pfs25M-EPA/AS01 (Phase I) and Pfs230D1M-EPA/AS01 (Phase II). The parasite's blood stage is linked to causing symptomatic/clinical malaria and disease transmission through the transfer of gametocytes from an infected human to vector *Anopheles*. In parallel with pre-erythrocyte and erythrocyte stage-based vaccines, sexual-stage or gametocyte surface antigen-targeting vaccines are used to treat malaria [142]. Targeting the sexual stage or gametocyte antigen prevents ookinete maturation and sporozoite development, release, and transmission. Still, it does not stop malaria symptoms in infected individuals. The antigens that are being targeted are ookinete surface protein Pfs25, a male gametocyte protein P48/45 (*P. vivax* Pvs48/45 and Pvs47), Pfs47, and gametocyte antigen Pf230 [143–146]. Cell-transversal protein for ookinete and sporozoite (CELTOS), an anticipated vaccine candidate required for ookinete transversal and sporozoite infection, has shown increased immunogenicity when adjuvanted with CpG or poly IC or both [147]; 25 kDa ookinete surface protein (Pfs25) fused with a complement inhibitor C4b-binding protein IMX313 encoded by ChAd63 and modified vaccinia virus Ankara (MVA) viral vector is a recombinant DNA or vectored vaccine candidate for transmission blocking [143]. Recombinant IMX313 protein acts as a carrier by creating heptamer with antigen and generating a solid antibody response [143]. Antibodies that respond to these antigens have been tested for blocking activity through a standard membrane-feeding assay (SMFA). A certain antibody titer level is required to secure the parasite's sexual stage progression in mosquitos. Pfs47 sexual stage antigen display on Acinetobacter Phage AP205 VLP elicited a robust transmission reducing activity (TRA) by antibody at a 5 µg/ml concentration purified from immunized mice [145]. In addition, considering mosquito midgut protein anopheline alanyl aminopeptidase N (AnAPN1) critical for traversal of parasite ookinete in mosquito midgut can be a proven potent strategy for transmission-blocking activity. A second-generation AnAPN1 vaccine construct containing crucial peptide epitopes adjuvanted with glucopyranosyl lipid adjuvant and saponin QS21 in liposomal formulation elicited antibody production [148].

### Recombinant DNA or viral vectored vaccine

DNA or vectored vaccine, ChAd63 MVA ME-TRAP (phase II), is a current vaccine technology to present intracellular antigens and induces a strong CD8 + mediated immune response with pro-inflammatory cytokine production required against malaria infection. Chimpanzee Adenovirus 63 (ChAd63) and MVA, a non-replicating viral vector encoding different malaria proteins, includes 25 kDa ookinete protein Pfs25, RH5, PvDBP, CSP, and multiepitope chain of TRAP (ME-TRAP) [143, 149–151]. Self-amplifying RNA vaccine is a novel vaccine technology that introduces an mRNA construct encoding an antigen *Plasmodium* macrophage migration inhibitory factor, a *Plasmodium* protein that can quench the host pro-inflammatory cytokines, and a replication machine for self-amplification [152]. This novel self-amplifying RNA vaccine will minimize the number of doses and reduce the declination of antibody titer over a while. Table 3 summarizes the malarial vaccines, approved or under clinical trial or investigation, along with their vaccine type, target antigen, and mode of action.

Vaccine delivery, antigen uptake, and accurate antigen presentation are crucial for a vaccine's efficiency. A self-assembling protein nanoparticle is a current strategy to present Th or Tc cell epitopes of CSP or other blood and sexual stage proteins [153, 154]. Synthetic or inorganic nanoparticles can be proved to be a safe and novel approach to delivering or presenting antigens without any negative impact in the murine model [155, 156]. A carrier protein is sometimes required to particulate the antigen in nano size and simultaneously works as an adjuvant. Exo-protein A from *Pseudomonas aeruginosa* and IMX313, a homolog of human complement four binding protein (C4bp), were tested for their reactogenicity and immunogenicity with different vaccine candidates. At the same time, Advax (delta inulin polysaccharide), a co-adjuvant with poly (I:C), increases the half-life of the antigen, resulting in persisting immune response [136, 143, 157].

### Diagnosis

According to National Institutes of Health, diagnosis is a complex process to identify a disease, illness, or injury by examining the signs and symptoms and comparing them to an existing set of categories that define a particular condition, as the medical profession agrees. Diagnosis is the distinguishing of a diseased condition from health, and it leads to the appropriate treatment and prognosis [158]. Diagnosis occurs at three levels: first, where a class of disease is determined (such as a cardiac disorder); second, the subject to be diagnosed is particularized (such as a 45-year-old male); third, a specific reasoned categorization is made (such as coronary artery disease) [158].

**Fig. 6:**
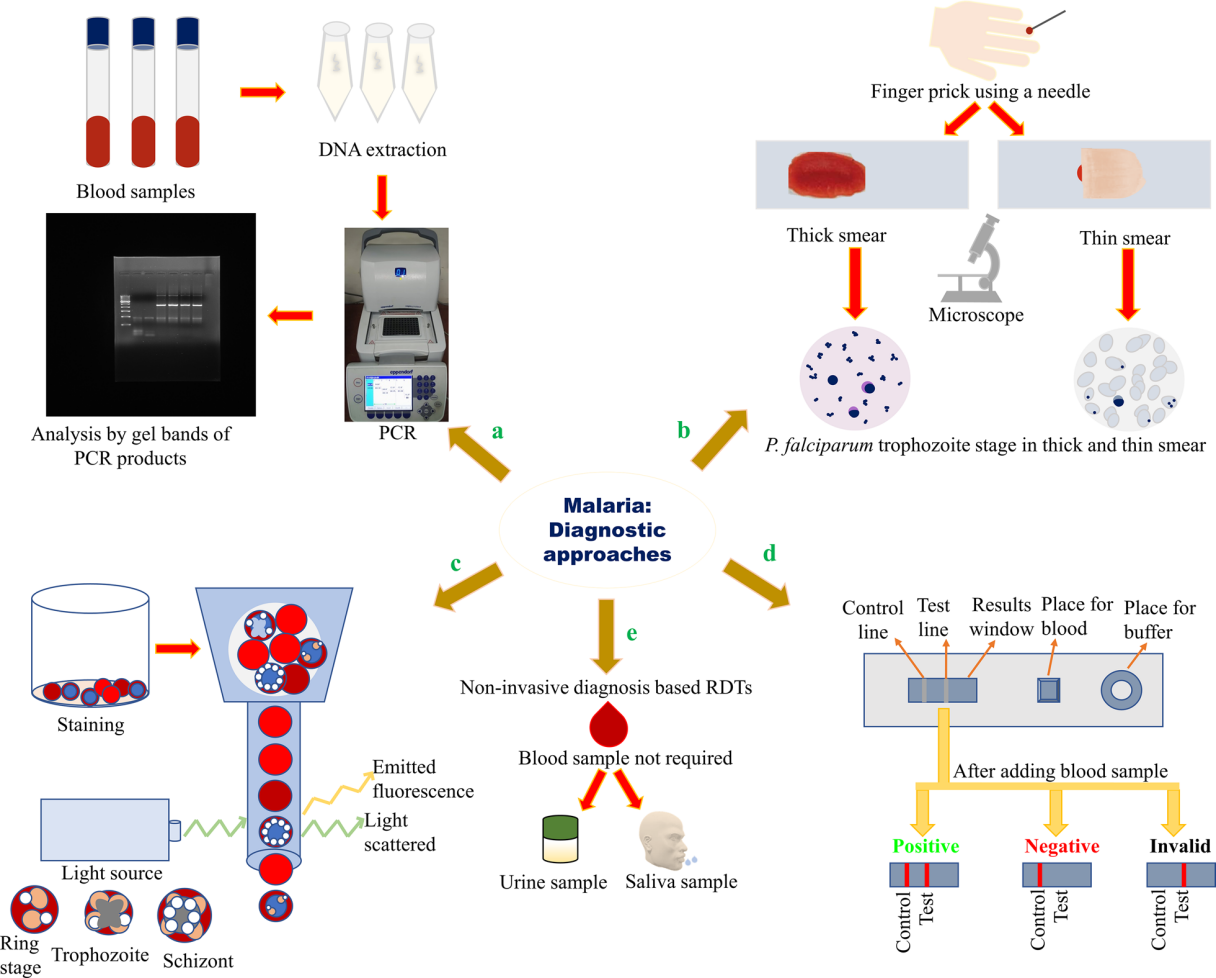
Diagnosis methods for malaria detection using (**a**) PCR. (**b**) Use of microscopy in malaria diagnosis: A blood sample is taken by pricking the finger, and two types of smears can be prepared: thick (for the presence of *Plasmodium*) and thin (for identification of species of *Plasmodium*). The figure shows how the trophozoite stage is visualized in thick smear and thin smear. **c** Flow cytometry in malaria diagnosis: Fluorochrome staining and analysis by flow cytometry. **d** Rapid diagnostic test (RDT) for malaria: Cassette and interpretation of the assay results as positive, negative, or invalid. (**e**) Diagnosis by RDTs using samples other than blood such as saliva and urine. PCR, polymerase chain reaction

The detection of malaria is essential at the initial stage. Otherwise, the disease might develop severe complications, especially *P. falciparum* infections, which may be fatal [159]. The review paper explains the available diagnostic methods, such as RDT, polymerase chain reaction (PCR), and microscopy (Fig. 6), to detect malaria parasite infection and their recent advancements.

### Diagnostic methods currently in use

#### Clinical diagnosis

This type of malaria diagnosis is based on the symptoms displayed by the infected individual. It is used for diagnosis in case of the unavailability of laboratory facilities or self-diagnosis [160, 161]. Like malaria, many diseases cause symptoms such as fever, headache, fatigue, and anemia at later stages. In ancient times, people found it clinically challenging to distinguish malaria from other fevers. Significantly, the two conditions (malaria and typhoid) were most likely to be confused [162]. Self-diagnosis and self-treatment are prone to errors [163]. Therefore, clinically suspected individuals should constantly be tested using the diagnostic tools available at the hospitals.

#### Microscopy

A famous German chemist and bacteriologist, Gustav Giemsa, introduced a mixture of methylene blue and eosin in 1904, after which Giemsa staining, followed by imaging, was used for malaria diagnosis [164]. Although advanced diagnostic methods and automatic devices are being developed, the microscopic examination of blood films remains the gold standard method.

Eosin and methylene blue are the two main components that make up the Giemsa staining solution from which eosin stains the parasite’s nucleus red. In contrast, methylene blue causes the cytoplasm to appear blue colored [165]. A thick blood film stained using Giemsa stain is usually used to determine the presence of parasites, whereas a Giemsa-stained thin blood film helps identify the species under light microscopy. Approximately 50 times more blood is examined in a thick blood film than in a thin blood film [165]. However, while staining, 60–80% of parasites may be lost in the case of Giemsa-stained thick blood film [166]. The inexpensive Giemsa microscopy differentiates the *Plasmodium* species and quantifies the parasites.

Since mature erythrocytes do not contain DNA or RNA, whereas the parasites do, fluorescent dyes such as acridine orange are used to detect the *Plasmodium* parasites. For this, the patient’s blood sample is incubated with acridine orange, staining the DNA and RNA of different developmental stages of *Plasmodium* [167, 168]. The fluorescent parasites are then observed using a conventional fluorescence microscope or a fluorescence microscope based on LED [167, 168]. A fluorescent microscope with an interference filter was developed to image the thin blood films stained with acridine orange [169]. Although this method is feasible, trained personnel must correctly label the patient’s blood sample and have the expertise to read the slides.

#### Rapid diagnostic test

RDT is a device that detects the malaria parasite and proves to be an important alternative in cases where there is a lack of a microscope or time to scan the blood films [170]. It is a simple and fast diagnostic tool that detects the parasite in a small amount of blood sample (5–15 µl) by immunochromatographic assay, which involves monoclonal antibodies against the parasite’s antigen [171]. Currently, the available RDTs detect HRP2 (histidine-rich protein), pLDH (parasite lactate dehydrogenase), and aldolase [170]. They are more than 95% sensitive in *P. falciparum* infections, but this sensitivity level has not yet been achieved for non-*P. falciparum* infections [171]. The RDTs are available in different formats, such as dipsticks, cards, and cassettes [172].

Dipsticks have been used worldwide to detect malaria antigens in the blood. These rapid immuno-chromatographic tests (based on detecting the circulating antigens) are specific to the parasite with the help of specific antibodies attached to a membrane [173]. Dipstick tests were initially used to detect *P. falciparum* infections only as they target HRP2 expressed by *P. falciparum* during the trophozoite stage [174, 175]. Examples include Parasight-F, ICT Malaria P.f., and PATH Falciparum Malaria IC Strip [176]. However, newer tests, such as the OptiMAL assay and the ICT Malaria P.f/P.v assay, can detect *P. falciparum* and *P. vivax* infections. Both tests can also differentiate between the two species. The OptiMAL assay is based on pLDH detection, and these two parasitic species show antigenic differences in their pLDH isoenzymes [177]. The ICT Malaria P.f/P.v assay targets HRP2 of *P. falciparum* and *P. vivax* [178]. The dipsticks are simple to use and handle. They can be used for malaria self-testing, so travelers and tourists visiting malaria-endemic regions are advised to carry them [173]. In febrile cases, they can use these test kits when they cannot reach adequate professional help in time [179].

Cards and cassettes are safer to use than dipsticks as they can prevent blood contamination, but the disadvantage lies in their cost, which is 40% higher than for dipsticks. Another issue is that they are more time-consuming than dipsticks. Most available cards and cassettes target two antigens: HRP II/pLDH, HRP II/pan pLDH, or HRP II/pan aldolase [172].

#### Polymerase chain reaction

PCR-based tests have improved the limit for detection of malaria infection with < 0.02 parasites/µl [180, 181]. Although PCR detects cases with low parasitemia, it has been observed that it may miss some cases even with high parasitemia [182]. The PCR uses thermostable DNA polymerases of bacterial origin and amplifies even tiny fragments of DNA by using different temperatures at different stages of the cycle. Several PCR approaches are used to detect *Plasmodium* infection.

Nested PCR involves two consecutive rounds of amplification with two sets of primers. The first amplification product is used as the template for the second round, in which species-specific primers are used [183, 184]. For the first reaction, the primers *rPLU1* and *rPLU5* are used for the amplification of the genomic DNA of *Plasmodium*; for detecting *P. falciparum*, the products of the first reaction are then amplified using *rFAL1* and *rFAL2*, *rVIV1* and *rVIV2* for *P. vivax*, *rOVA1* and *rOVA2* for *P. ovale*, and *rMAL1 and rMAL2* for *P. malariae* [185]. Furthermore, the PCR products are separated by running agarose gel electrophoresis and stained with ethidium bromide, followed by visualization under UV light to check which lanes contain products positive for malaria [186].

Real-time PCR or qPCR is used for real-time observation of the replication and amplification process [184]. Fluorescent labels such as SYBR green, sequence-specific oligonucleotide probes, and photo-induced electron transfer fluorogenic primers are used to monitor the amplicon formation [187–189]. This is based on the principle that their fluorescence intensity is closely related to the number of amplification products [184].

Direct PCR assays are also available for *Plasmodium* detection. In most forms of PCR, DNA extraction from blood samples is a crucial step, but direct PCR bypasses DNA extraction [190]. Therefore, the time, cost, and labor required to get DNA is reduced, but this procedure might miss the asymptomatic infections because of relatively low parasitemia [184]. Phusion blood direct PCR kit (Thermo Scientific, Waltham, MA) has been used to perform direct PCR using dried blood spots as the sample [191].

Reverse-transcriptase PCR allows the targeting of expressed RNA sequence instead of the gene, which enables the determination of *Plasmodium* in its specific stages [184]. A real-time reverse-transcriptase PCR was developed to detect *Plasmodium* by amplifying the RNA and DNA of 18S rRNA genes [192]. It can detect infections with parasitemia as low as 0.002 parasite/μl [184].

#### Flow cytometry

During the life cycle of *Plasmodium*, the parasites invade the RBCs and further grow and multiply in these cells. Therefore, these stages cause clinical symptoms and are the targets for various drugs [193]. Also, the detection of the presence of *Plasmodium* in the blood is used to diagnose malaria for which flow cytometry has proved useful [193]. The analysis of the development of the blood stages by flow cytometry is reproducible and rapid [193, 194]. Flow cytometry is performed using fluorescent dyes specific to nucleic acids since RBCs do not contain DNA. Any DNA-specific fluorescence detected in the RBC population results from the fluorescent dyes bound to *Plasmodium* DNA [193]. Therefore, infected cells can be differentiated from non-infected cells, and this method can even be used to determine the parasite’s developmental stage. As the plasmodia multiply in the RBCs, the stained parasites' fluorescence intensity increases during their development [193]. It is a sophisticated approach to diagnosing malaria, but the equipment is expensive and requires trained personnel for operation and maintenance, which would affect the accuracy severely [170].

#### Automated blood cell analyzers

These can detect parasites at low parasitemia levels, such as 5–20 parasites/µl of blood, but light microscopy, if performed by an experienced pathologist, might detect even lower parasitemia levels [195]. Therefore, the automated blood analyzer is not appropriate as a screening test. Still, it plays a role in detecting additional cases, such as those with no clinical suspicion that lead to a specific request for a malaria test [196]. For determining the species of *Plasmodium* and parasitemia, microscopy is required as the automated blood cell analyzer, Abbott Cell-Dyn 3500, only lets one know about the presence of abnormal monocyte and neutrophil cell populations [197]. The instrument’s sensitivity is based on pigmentation. Therefore, early infections might not get detected because of the low abundance of malaria pigment in the initial stage [198]. New models with higher sensitivity for malaria detection have been developed [199].

#### Serological detection

The antibodies to *Plasmodium* may persist for months once they appear after the erythrocytes are invaded by the parasite [196]. This can be used to diagnose the presence of *Plasmodium* in the serum of the patients. The immunofluorescence antibody test has been used to detect *Plasmodium*-specific antibodies in serum samples [200]. The serum sample is applied to a slide on which *Plasmodium* antigen was prepared and stored at –30 ºC, followed by a quantitative result using fluorescence microscopy to determine the amount of IgG and IgM [200]. Enzyme-linked immunosorbent assay can also be used for antibody detection [201]. Although these two techniques are simple, they require more time and trained personnel [170].

#### Non-invasive diagnosis

Samples other than blood, such as body fluids (urine and saliva) or fecal matter or hair, represent an alternative as they are obtained without invasion, thus avoiding the pain associated with invasive procedures and the consequences of the social and cultural beliefs related to blood sampling, leading to an increase in the participation in mass screening programs [202, 203]. Techniques like PCR, immunoassay, microfluidics, and immunochromatography are used for *Plasmodium* detection in such samples [170].

### Recent developments in malaria diagnosis

**Fig. 7:**
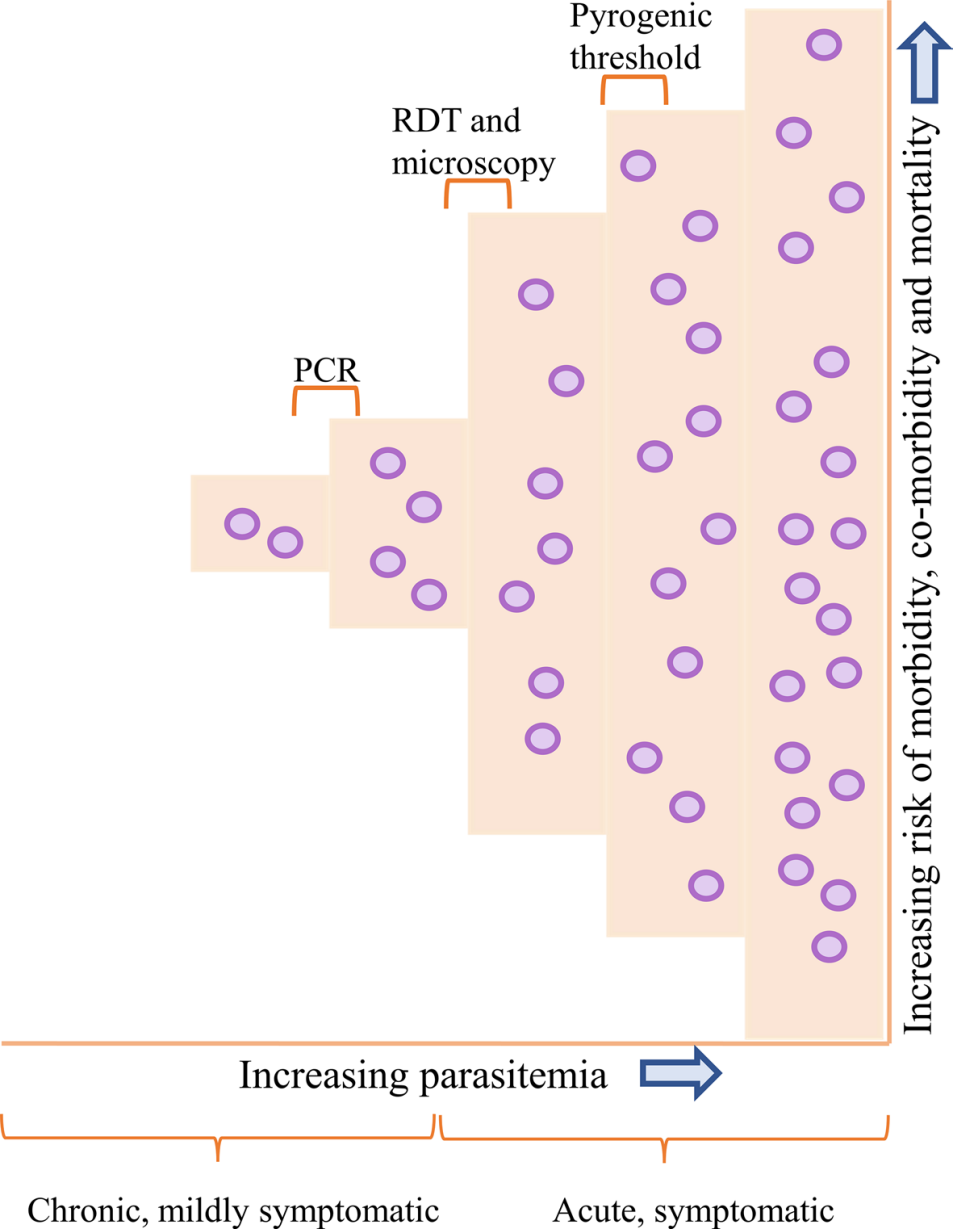
The range of malaria infection and a comparison of the sensitivity of three diagnostic methods: microscopy, RDT, and PCR. Microscopy and RDTs are sensitive enough to detect symptomatic infections but not those with low parasite density. PCR is more sensitive than the other two techniques (RDT and microscopy) but is unable to detect infections with very low parasitemia. PCR, polymerase chain reaction; RDT, rapid diagnostic test

**Fig. 8:**
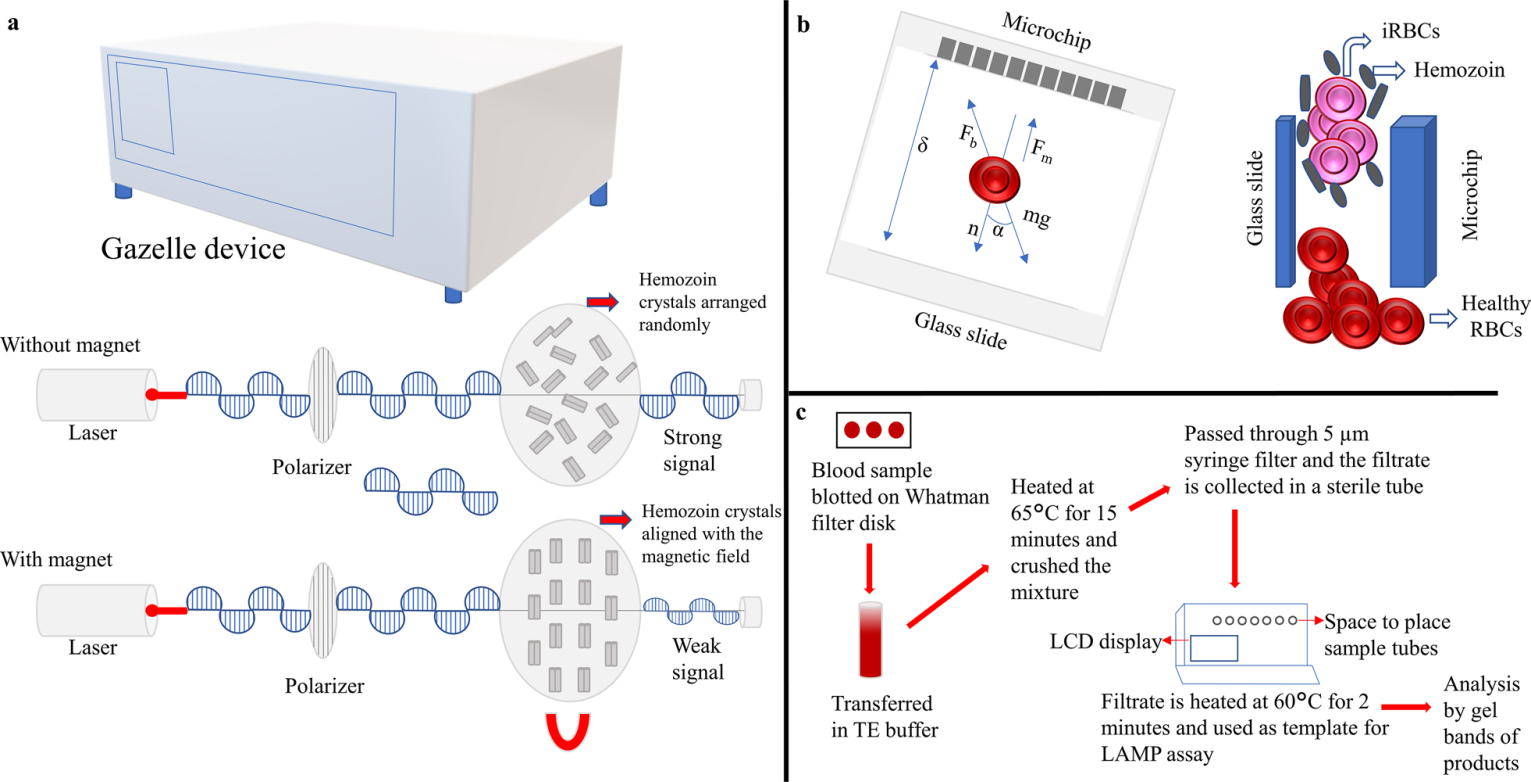
Future tools for malaria diagnosis. **a** Gazelle device and its mechanism based on magneto-optical detection. **b** TMek: The iRBCs and hemozoin crystals get captured on the cylindrical Ni concentrators of microchip and the healthy RBCs are sedimented. (**c**) LAMP for malaria diagnosis: Procedure for DNA extraction and LAMP assay. TMek, Tid Mekii; iRBC, infected red blood cell; LAMP, loop-mediated isothermal amplification; DNA, deoxyribonucleic acid

Malaria detection is a crucial step for proper and timely treatment. Moreover, the two most commonly used malaria diagnostic approaches, microscopy and RDT, can diagnose symptomatic infections but are not sensitive enough to detect low-parasitemia asymptomatic infections. This means that even if asymptomatic people reach the health centers for diagnostic examination, they will probably remain undiagnosed. The PCR can detect disease with lower parasitemia, but those with very low density cannot be detected (Fig. 7) [204]. Several recently developed and emerging techniques seem promising. However, they still have a few limitations regarding detecting certain parasite stages, and discrimination between different stages is also a concern [205]. For example, the detection of dormant stages or hypnozoites of *P. vivax* and *P. ovale* cannot be done with the currently available tools [205]. Among the recently developed techniques for malaria diagnosis, one is loop-mediated isothermal amplification, a molecular approach based on the amplification of nucleic acid during which specific genes are converted to loops so that continuous amplification can occur (Fig. 8) [205, 206]. Gazelle is a new device for malaria diagnosis based on detecting malaria pigment, hemozoin. It quickly detects hemozoin particles in the blood sample and is cost-effective (Fig. 8) [207]. Since hemozoin contains iron components, the magnetic field aligns the particles. An internal light source shines a light on the sample, and the measurement is made of the amount of transmitted light in both the presence and absence of a magnetic field [207]. In its absence, the hemozoin particles become randomly oriented because of Brownian motion, while in a magnetic field, the particles become aligned and block light transmission [207]. Since all five species produce hemozoin, this device allows the detection of malaria caused by all five species. A non-invasive, rapid technique based on near infrared spectroscopy has been developed recently, which can diagnose malaria due to *P. falciparum* and *P. vivax* through the skin of malaria patients. It uses only a hand-held spectrometer, thus making it a reagent-free technique; this miniature spectrometer is used to shine near-infrared light on the ear, arm, or finger of the individual, and spectra are generated, which are then used for making predictions using machine learning algorithms. The study in Brazil showed 92% accuracy for the arm and 93% predictive accuracy for differentiating between *P. falciparum* and *P. vivax*. The bands observed in the spectra enable the identification of positive and negative malaria cases and are mainly due to hemozoin [208].

### Anticipated future developments

Many efforts are being made worldwide to understand the parasite *Plasmodium* and the *Anopheles* mosquito to control malaria successfully. The massive number of malaria cases and deaths distress the African region, tropical countries, and many others. Over the last century, efforts have been made to eradicate malaria globally. However, there are several roadblocks to eliminating malaria, including our understanding of the biology of the malaria parasites, the complex life cycle, and the parasite’s immune evasion. Simultaneously, the parasite has evolved drug resistance against most of the available antimalarial drugs, including WHO-recommended ACT. There is an urgent need to find novel antimalarials with the help of the latest strategy such as computer-aided drug design, fragment-based drug design, high-throughput screening, and drug repurposing approach. Apart from drug development, it has increased the need for an effective vaccine to eliminate malaria. No malaria vaccine can generate 100% sterile protection to achieve WHO's 90% malaria eradication goal by 2030. However, with low efficacy and subsequent antibody decline with time, the world has its first malaria vaccine, RTS,S (Mosquirix).

Following RTS,S, its second-generation vaccine R21 with improved efficacy is also moving forward quickly. The major obstacle in developing immunogenic malaria vaccines is the parasite's multi-stage life cycle, antigenic variation and polymorphisms, limited choice of immunogen, and vaccine candidate evaluation. Efforts can be made to remove these obstacles, and further steps should be taken to improve the efficacy of the existing RTS,S vaccine. This can be done by introducing better adjuvants and new technologies to present more and more antigens to the host immune system. *Qubevirus durum* bacteriophage and SpyTag/SpyCatcher system are a few to multimerize the antigen on the surface of a VLP [209, 210]. Bacterial vector vaccines expressing pathogen proteins and antigen-presenting cells are new tools under investigation that can be considered for malaria. However, it is too early to know the efficiency of these vaccine technologies [211]. Multiple anticipated vaccine candidates still need to be considered for improvement and newer technologies.

Apart from the treatment, a timely and accurate diagnosis of malaria is an important event that leads to saving humans from malaria disease. Malaria diagnosis is one of the effective strategies for disease management since it is curable if diagnosed promptly. Although many developments and newer techniques are continuously emerging, microscopy remains the gold standard. Several methods are better than microscopy in terms of accuracy or sensitivity. The methods discussed above have certain advantages and disadvantages, and it cannot be determined which is the best or most appropriate among them. It is unknown whether there is an ideal diagnostic method for malaria that is simple, accurate, quick, affordable, easy to handle, and painless. So, the search continues, and we have high hopes for new ideas and techniques. Currently, microscopy and RDTs are the most commonly used methods to diagnose malaria. Still, they will not be sufficient as the world moves towards malaria elimination, and newer techniques are required, enabling mass screening for asymptomatic infections, the surveillance of continued transmission, and the management of symptomatic infections [204, 212]. One such diagnostic technique that has been developed is known as TMek, a lab-on-a-chip diagnostic method that directly quantifies the level of parasitemia (Fig. 8) [213]. It exploits the magnetic properties of hemozoin (malaria pigment) nanocrystals and provides the quantification value in 10 min [213]. Also, the laser-based non-invasive method of malaria detection is an excellent step in the pain-free detection of malaria. It may be developed into a handy instrument to screen large populations without a sophisticated setup, similar to the infrared thermometer.

## Conclusion

Malaria is an infectious disease affecting people globally. Without prompt diagnosis and treatment, the condition can worsen, which is why proper diagnosis and treatment are essential. Overall, the progress in eliminating malaria globally has been satisfactory, and the number of malaria cases has declined [214]. Also, the available therapeutics in the form of drugs and vaccine have played a significant role in restraining malaria aftermaths. The presence of diagnostic approaches for malaria detection has also made it possible to reach the last corner of society. But still, diagnosis of asymptomatic malaria and removal of false-positive results even after a recovery has remained the bottleneck for accurate diagnosis for containing the spread of malaria. Moreover, the lack of a highly efficient vaccine has also hampered malaria infection preventive measures, especially in African regions. In addition, the growing concern about parasite resistance to the available medicines has worsened the scenario. Therefore, it is difficult to assess how close we are to the 2021 theme of malaria day, i.e. 'Zeroing in on malaria elimination.' Hence, we must align with the 2022 theme of malaria day by focusing on new and innovative approaches to reduce malaria's burden and save lives.

## Data Availability

Not applicable.

## Abbreviations

ACT: Artemisinin combination therapy
AMA: Apical membrane antigen
CELTOS: Cell traversal protein for ookinete and sporozoites
CHMI: Controlled human malaria infection
CSP: Circumsporozoite protein
GAP: Genetically attenuated parasite
GTS: Global technical strategy
HRP: Histidine-rich protein
LAMP: Loop-mediated isothermal amplification
MSP: Merozoite surface protein
PCR: Polymerase chain reaction
Pf: *Plasmodium falciparum*
PfSPZ: *Plasmodium falciparum* sporozoite
PfSPZ-CVac: *Plasmodium falciparum* sporozoite chemoprophylaxis vaccine
Pv: *Plasmodium vivax*
PvDBP: *Plasmodium vivax* duffy binding protein
RBC: Red blood cells
RDTs: Rapid diagnostic tests
RH5: Reticulocyte binding protein homolog 5
SMFA: Standard membrane feeding assay
TBV: Transmission blocking vaccine
TLR: Toll-like receptor
VLP: Virus-like particle
WHO: World Health Organization
